# Smoke-Free and Tobacco-Free Policies in Colleges and Universities ― United States and Territories, 2017

**DOI:** 10.15585/mmwr.mm6724a4

**Published:** 2018-06-22

**Authors:** Teresa W. Wang, Michael A. Tynan, Cynthia Hallett, Laura Walpert, Maggie Hopkins, Darryl Konter, Brian A. King

**Affiliations:** ^1^Office on Smoking and Health, National Center for Chronic Disease Prevention and Health Promotion, CDC; ^2^American Nonsmokers’ Rights Foundation, Berkeley, California.

Each year in the United States, cigarette smoking causes an estimated 480,000 deaths, including approximately 41,000 deaths from secondhand smoke exposure among nonsmoking adults (*1*). Smoke-free policies protect nonsmokers from secondhand smoke exposure, reduce the social acceptability of smoking, help in preventing youth and young adult smoking initiation, and increase smokers’ efforts to quit smoking (*1*,*2*). Given that 99% of adult cigarette smokers first start smoking before age 26 years and many smokers transition to regular, daily use during young adulthood (*2*),* colleges and universities represent an important venue for protecting students, faculty, staff members, and guests from secondhand smoke exposure through tobacco control policies (*3*). To assess smoke-free and tobacco-free policies in U.S. colleges and universities, CDC and the American Nonsmokers’ Rights Foundation (ANRF) determined the number of campuses nationwide that completely prohibit smoking (smoke-free) or both smoking and smokeless tobacco product use (tobacco-free) in all indoor and outdoor areas. As of November 2017, at least 2,082 U.S. college and university campuses had smoke-free policies. Among these campuses, 1,743 (83.7%) were tobacco-free; 1,658 (79.6%) specifically prohibited electronic cigarette (e-cigarette) use; and 854 (41.0%) specifically prohibited hookah smoking. Smoke-free and tobacco-free policies on college and university campuses can help reduce secondhand smoke exposure, tobacco use initiation, and the social acceptability of tobacco use (*1*–*3*).

Data on smoke-free and tobacco-free policies enacted as of November 2017 were obtained from ANRF’s College Campus Tobacco Policy Database,^†^ the only national repository of tobacco restrictions on college campuses in the United States. The database is compiled using a daily news digest from Internet searches, as well as direct communication with state and local health departments, university officials, students, and alumni.^§^ The policies then are analyzed using standardized criteria and entered into the database. Campuses eligible for consideration are located in all 50 U.S. states, the District of Columbia, commonwealths, territories, and in tribal entities.

College and university campuses were considered smoke-free if they completely prohibited smoking in all indoor and outdoor areas, and tobacco-free if they prohibited both smoking and smokeless tobacco product use in all indoor and outdoor areas.^¶^ In addition, those that explicitly prohibited use of e-cigarettes and hookah smoking were also assessed.** For institutions comprising multiple physical learning sites with or without distinct policies, each site was evaluated as a separate campus. Campuses without smoke-free or tobacco-free policies were not included in the database, and data on the total number of U.S. college and university campuses as defined in the context of this report were unavailable. Therefore, it was not possible to summarize the number of smoke-free campuses as a percentage of total U.S. campuses. Findings were reported overall and by state and campus type (public; private; community college; historically black college or university; and tribal).^††^ Campus type categories were not mutually exclusive, and campuses could be categorized as multiple types.

As of November 2017, at least 2,082 U.S. college and university campuses were smoke-free (Table 1). Among these campuses, 1,743 (83.7%) were tobacco-free; 1,658 (79.6%) specifically prohibited e-cigarette use; and 854 (41.0%) specifically prohibited hookah smoking.

**TABLE 1:** College and university campuses* with smoke-free policies,^†^ tobacco-free policies,^§^ and policies specifically prohibiting e-cigarette use and hookah smoking, by campus type — United States and territories, 2017

Type of campus^¶^	No. of smoke-free campuses	Campuses with additional policies**		
		Tobacco-free no. (%)	E-cigarettes no. (%)	Hookah no. (%)
Public	1,616	1,375 (85.1)	1,373 (85.0)	692 (42.8)
Community college	1,209	1,066 (88.2)	1,018 (84.2)	459 (38.0)
Private	448	350 (78.1)	283 (63.2)	159 (35.5)
Historically black	58	42 (72.4)	37 (63.8)	28 (48.3)
Tribal	18	18 (100.0)	2 (11.1)	3 (16.7)
**Total**	**2,082**	**1,743 (83.7)**	**1,658 (79.6)**	**854 (41.0)**

* Institutions comprising multiple campuses or sites, with or without distinct policies, are counted separately.

^†^ As of November 2017, the campus is covered by a law or policy that prohibits smoking (at minimum) in all indoor and outdoor areas. The only exemptions include one’s personal vehicle, research in a controlled laboratory setting, or religious ceremonial purposes. Smoke-free campuses covered by state law are not indicated separately from campuses covered by institutional policies.

^§^ As of November 2017, the campus is covered by a law or policy that prohibits smoking and smokeless tobacco use in all indoor and outdoor areas. The only exemptions include one’s personal vehicle, research in a controlled laboratory setting, or religious ceremonial purposes. Tobacco-free campuses covered by state law are not indicated separately from campuses covered by institutional policies.

^¶^ College and university campus types were not mutually exclusive. Campuses could be categorized as multiple campus types and counted more than once (e.g., private and community college) and therefore could sum to more than the total. A public college or university was defined as a campus funded by government means. A private college or university was defined as a campus not funded by government means. A community college was defined as a campus with “community college” in the name, or described itself as one in the documentation encountered during analysis, or a reliable source confirmed this status. A historically black college or university was defined as a campus that described itself as one in the documentation encountered during analysis or a reliable source has confirmed this status. A tribal college or university was defined as a campus on American Indian/Alaska Native sovereign land.

** Indicated as a subset or percentage of smoke-free campuses.

A total of 1,616 public college and university campuses were smoke-free. Among these public campuses, 1,375 (85.1%) were tobacco-free; 1,373 (85.0%) specifically prohibited e-cigarette use; and 692 (42.8%) specifically prohibited hookah smoking. Among the 448 private campuses with smoke-free policies, 350 (78.1%) were tobacco-free; 282 (63.2%) specifically prohibited e-cigarette use; and 159 (35.5%) specifically prohibited hookah smoking. Among the 1,209 community college campuses with smoke-free policies, 1,066 (88.2%) were tobacco-free; 1,018 (84.2%) specifically prohibited e-cigarette use; and 459 (38.0%) specifically prohibited hookah smoking. Among the 58 historically black college or university campuses with smoke-free policies, 42 (72.4%) were tobacco-free; 37 (63.8%) specifically prohibited e-cigarette use; and 28 (48.3%) specifically prohibited hookah smoking. Among the 18 tribal campuses with smoke-free policies, all 18 were tobacco-free; two (11.1%) specifically prohibited e-cigarette use; and three (16.7%) specifically prohibited hookah smoking. By state or territory, the number of college and university campuses with a smoke-free policy ranged from one in Hawaii and the Northern Mariana Islands to 108 in California and North Carolina (Table 2).

**TABLE 2:** Distribution of college and university campuses* with smoke-free policies^†^ and tobacco-free policies^§^ — United States and territories, 2017

State/Territory	Campus type	
	Smoke-free no.	Tobacco-free^¶^ no. (%)
Alabama	45	39 (86.7)
Alaska	6	6 (100.0)
Arizona	42	42 (100.0)
Arkansas**	60	26 (43.3)
California	108	85 (78.7)
Colorado	11	8 (72.7)
Connecticut	6	3 (50.0)
Delaware	9	9 (100.0)
Florida	85	70 (82.4)
Georgia	60	58 (96.7)
Hawaii	1	1 (100.0)
Idaho	13	8 (61.5)
Illinois**	95	23 (24.2)
Indiana	71	65 (91.5)
Iowa**	104	58 (55.8)
Kansas	30	20 (66.7)
Kentucky	92	87 (94.6)
Louisiana**	91	86 (94.5)
Maine	26	26 (100.0)
Maryland	24	22 (91.7)
Massachusetts	29	16 (55.2)
Michigan	71	69 (97.2)
Minnesota	30	29 (96.7)
Mississippi	38	34 (89.5)
Missouri	55	50 (90.9)
Montana	8	8 (100.0)
Nebraska	19	19 (100.0)
Nevada	3	0 (0.0)
New Hampshire	6	4 (66.7)
New Jersey	36	27 (75.0)
New Mexico	2	1 (50.0)
New York	98	81 (82.7)
North Carolina	108	104 (96.3)
North Dakota	12	12 (100.0)
Ohio	46	44 (95.7)
Oklahoma	56	56 (100.0)
Oregon	32	27 (84.4)
Pennsylvania	68	57 (83.8)
Rhode Island	2	2 (100.0)
South Carolina	68	63 (92.6)
South Dakota	25	21 (84.0)
Tennessee	40	33 (82.5)
Texas	89	86 (96.6)
Utah	3	3 (100.0)
Vermont	25	25 (100.0)
Virginia	4	4 (100.0)
Washington	21	20 (95.2)
West Virginia	16	16 (100.0)
Wisconsin	90	87 (96.7)
Wyoming	NI	NI
American Samoa	NI	NI
Guam	2	2 (100.0)
Marshall Islands	NI	NI
Micronesia	NI	NI
Northern Mariana Islands**	1	1 (100.0)
Palau	NI	NI
Puerto Rico	NI	NI
Virgin Islands	NI	NI
**Total**	**2,082**	**1,743**

**Abbreviation:** NI = none identified.

* Institutions comprising multiple campuses or sites, with or without distinct policies, are counted separately.

^†^ As of November 2017, the campus is covered by a law or policy that prohibits smoking (at minimum) in all indoor and outdoor areas. The only exemptions include one’s personal vehicle, research in a controlled laboratory setting, or religious ceremonial purposes. Campuses that do not qualify as smoke-free under these definitions are not assessed.

^§^ As of November 2017, the campus is covered by a law or policy that prohibits smoking and smokeless tobacco use in all indoor and outdoor areas. The only exemptions include one’s personal vehicle, research in a controlled laboratory setting, or religious ceremonial purposes.

^¶^ Indicated as a subset or percentage of smoke-free campuses.

** Four states (Arkansas, Illinois, Iowa, and Louisiana) and the Northern Mariana Islands have enacted laws requiring comprehensive smoke-free indoor and outdoor public campuses. Iowa’s smoke-free campus provision applies to both public and private institutions. Campuses covered by state law are not indicated separately from campuses covered by institutional policies.

## Discussion

In September 2012, the U.S. Department of Health and Human Services, the University of Michigan, and the American College Health Association collaboratively launched the Tobacco-Free College Campus Initiative to promote and support the voluntary adoption and implementation of tobacco-free policies at universities, colleges, and other institutions of higher learning across the United States. At the time, 774 colleges and universities were identified as having a smoke-free campus policy, 562 (72.6%) of which were tobacco-free.^§§^ The findings from this study indicate that, as of November 2017, the number of campuses with smoke-free or tobacco-free policies had risen to 2,082 and 1,743, respectively, suggesting that the number of U.S. college and university campuses with such policies has more than doubled over the past half-decade. Smoke-free and tobacco-free policies at colleges and universities can help reduce secondhand smoke exposure, tobacco use initiation, and the social acceptability of tobacco use (*1*–*3*).

These results include campuses that might be smoke-free or tobacco-free because of policies at the institutional, local, state, or territorial levels. *Healthy People 2020* objective TU-13.17 monitors the number of states and the District of Columbia that have enacted laws that prohibit smoking on college and university campuses.^¶¶^ As of 2017, four states (Arkansas, Illinois, Iowa, and Louisiana) and the Northern Mariana Islands have enacted laws requiring smoke-free policies that prohibit smoking in all indoor and outdoor areas of public college campuses (*4*,*5*). Among these smoke-free laws, Arkansas’s law specifically prohibits e-cigarettes, Illinois’s law specifically prohibits e-cigarettes and hookahs, and the Northern Mariana Islands’ law specifically prohibits e-cigarettes and smokeless tobacco.*** Iowa’s smoke-free campus law is the only state law that extends to campuses at both public and private institutions.^†††^Given the evolving U.S. tobacco product landscape, addressing the diversity of tobacco products available on the market is important in the development of tobacco-free policies, including emerging products such as e-cigarettes and hookahs.

Because nearly all adult cigarette smokers begin smoking by young adulthood (*2*), colleges and universities can serve an important role in preventing tobacco product use initiation among nonusers, while also protecting students, faculty, staff members, and guests from secondhand smoke exposure. In 2015, approximately 40% of U.S. adults aged 18–24 years (12.6 million) were enrolled in 4,562 degree-granting post-secondary institutions (*6*),^§§§^ and a substantial proportion of young adults currently use at least one tobacco product: in 2015, one in five adults aged 18–24 years (21.4%) reported using a tobacco product some days or every day (*7*). Moreover, 23.8% of adults who attained some college education, but received no diploma, reported current use of at least one tobacco product (*7*). Given the trajectories of tobacco product use and initiation among young adults, interventions targeted toward this population, including tobacco-free and smoke-free policies in colleges and universities, might help accelerate efforts to reduce tobacco product use among young persons (*1*,*2*).

This study is subject to at least four limitations. First, these data might include policies that have been formally adopted but are not yet in effect. Second, whereas ANRF’s database is the only national repository of smoke-free campus policies, these policies are not collected systematically from all campuses in the United States and therefore might not contain all policies that currently exist. Third, ANRF’s database does not capture the total number of U.S. college and university campuses; comparable data would be needed to present the percentage of U.S. campuses with smoke-free or tobacco-free policies and to estimate the percentage of students protected. Finally, there is no uniform method for ascertaining how rigorously these policies are enforced. Previous research suggests that although tobacco-free campuses have increased in recent years, policy restrictiveness, implementation, and enforcement vary (*8*).

The U.S. Surgeon General has concluded that there is no risk-free level of secondhand smoke (*9*), and the public health benefits of smoke-free policies are well established in the scientific literature (*1*). Smoke-free and tobacco-free campuses can promote the health and well-being of a diverse intersection of students, faculty, staff members, and guests by protecting nonusers from the harmful effects of secondhand tobacco product emissions, reducing the social acceptability of tobacco product use, preventing tobacco use initiation, and promoting cessation (*1*,*2*,*9*). Continued efforts to monitor, promote, implement, and enforce smoke-free and tobacco-free policies in U.S. colleges and universities, in coordination with continued implementation of proven population-based interventions and tobacco product regulation (*10*), can help reduce the burden of tobacco product use among those who learn, live, work, and gather in these environments (*1*,*2*,*9*).

**Summary**

**What is already known about this topic?**

Cigarette smoking causes an estimated 480,000 U.S. deaths annually, including 41,000 from secondhand smoke exposure. Nearly all adult cigarette smokers start smoking before age 26 years, making smoke-free and tobacco-free policies at colleges and universities important.

**What is added by this report?**

As of November 2017, at least 2,082 U.S. colleges and universities had smoke-free policies, twice as many as in 2012. Among these campuses, 1,743 (83.7%) had tobacco-free policies and some specifically prohibited electronic cigarette use (1,658 [79.6%]) and hookah smoking (854 [41.0%]).

**What are the implications for public health practice?**

Efforts to monitor, promote, implement, and enforce smoke-free and tobacco-free policies in U.S. colleges and universities can help reduce the prevalence of tobacco product use and secondhand smoke exposure among those who learn, live, work, and gather in these environments.

## Footnotes

* https://www.ncbi.nlm.nih.gov/books/n/nap18997/pdf.

† https://no-smoke.org/colleges-universities-list-criteria/.

§ Information on school policies is also gathered by ANRF program personnel providing technical assistance in this area. Details are confirmed by reviewing a copy of the final policy, either on the institution's website or via an institution contact with access to the policy documentation.

¶ For smoke-free campuses, the law or institutional policy prohibits smoking of combustible tobacco products on the entire campus property, both indoors and out, including remote parking lots, stadiums, theater performances, and residential housing (where applicable). For tobacco-free campuses, the law or institutional policy prohibits smoking of combustible tobacco products and the use of smokeless tobacco products on the entire campus property, both indoors and out, including remote parking lots, stadiums, theater performances, and residential housing (where applicable). The only possible exemptions for smoke-free and tobacco-free campuses include one’s personal vehicle, research in a controlled lab setting, or religious ceremonial purposes.

** Coverage of hookah smoking is not consistently addressed under smoke-free policies for college or university campuses. Therefore, subsequent analyses determined the proportion of smoke-free policies that contain language specifically prohibiting hookah (e.g. “hookah,” “shisha,” or “water pipe”) use anywhere on campus.

†† A public college or university was defined as a campus funded by government means. A private college or university was defined as a campus not funded by government means. A community college was defined as a campus with “community college” in the name or that described itself as one in the documentation encountered during analysis, confirmed via a reliable source familiar with the entity or via Internet research. A historically black college or university was defined as a campus that described itself as one in the documentation encountered during analysis, confirmed via a reliable source familiar with the entity, or via Internet research. A tribal college or university was defined as a campus on American Indian/Alaska Native sovereign land.

§§ http://no-smoke.org/wp-content/uploads/pdf/smokefreecollegesuniversities-Jul-1-2012.pdf.

¶¶ http://www.healthypeople.gov/2020/topics-objectives/topic/tobacco-use/objectives.

*** Arkansas Clean Air on Campus Act 734 of 2009 (effective August 1, 2010), subsequently amended effective July 22, 2015, and Arkansas Medical Marijuana Amendment of 2016 (effective March 29, 2017); Illinois Smoke-Free Campus Act, 110 ILCS 64/1-99 (effective January 1, 2015); Commonwealth of the Northern Mariana Islands Smoke-free Air, PL 16-46, Act of 2008 (effective September 29, 2009).

††† Iowa Smoke-Free Air Act of 2008, Iowa Code Chapter 142 D (effective July 1, 2008).

§§§ Includes only institutions reporting enrollment data in Fall 2015. The number of degree-granting postsecondary institutions reported by the National Center for Education Statistics is not directly comparable to the number of smoke-free campuses identified in this report.
